# Author’s response to letter “Antimicrobials administration time in patients with suspected sepsis: faster is better for severe patients”

**DOI:** 10.1186/s40560-020-00472-1

**Published:** 2020-07-23

**Authors:** Johana Ascuntar, Deibie Mendoza, Fabián Jaimes

**Affiliations:** 1 GRAEPIC—Clinical Epidemiology Academic Group (Grupo Académico de Epidemiología Clínica), The University of Antioquia, Medellín, Colombia; 2 University of Antioquia, Medellín, Colombia; 3 Department of Internal Medicine, Universidad de Antioquia, Medellín, Colombia

## Abstract

We are appreciative to Dr. Jouffroy and Pr. Vivien for their responses and insights, and we agree with their words about the controversial aspect timing to antibiotic administration. Nevertheless, we stand firmly that it is not just about the time of administration of antimicrobials, but the early recognition and the comprehensive approach to recognize the most severe patients.

## Main text

We have read with great interest the letter of Dr. Jouffroy and Pr. Vivien. Their letter to the editor lists several concerns about our previously published article, which evaluated the effect on mortality of early antimicrobial administration in patients with sepsis [1]. Although we believe that these aspects have been mentioned in the article, it is important to return to them.

Our study is a secondary analysis of a prospective multicenter cohort. The first comments of the authors refer to our original study where our purpose was to estimate, through an instrumental analysis, the effect of each of the components of an early goal-directed therapy (EGDT) protocol, as well as the effect of the administration of antibiotics in the in-hospital mortality of patients with septic shock [2]. On this aspect, regarding the conclusions of the primary and not the secondary analysis, the authors mention several limitations related to the most recent definitions of septic shock. As we clarified in our article, at the time of recruitment and processing of the original study, the Sepsis-3 consensus had not yet been published [3], and therefore the previous definitions were used. Despite this, as we *present* in the article, we consider that the current recommendations for the early administration of antimicrobials within the first hour in patients with sepsis may represent a guideline that is not very applicable in a real emergency department setting. In this regard, we agree with the authors that a crowded emergency department would have logistical and time problems to ensure compliance with these benchmarks. This is precisely one of our conclusions when we affirm that a more comprehensive approach to sepsis and its treatment is needed, beyond just the simple time to antimicrobial administration, which considered as the simple unique intervention, could conduce only to the indiscriminate use of them.

In our article, we mentioned the low overall mortality rate (11.5%) as one of the limitations. Regarding this, it is necessary to clarify that we carried out an analysis by subgroups, and one of these was conformed by patients with septic shock, in which we applied the new definition proposed in the Sepsis-3 consensus. For this sub-population, the mortality rate was 33.5% (*n* = 60) and although the overall mortality rate is lower than expected; there are studies with mortality rates of 8.8% that describe associations of increased in-hospital mortality for each hour of delay in the administration of antimicrobials [4]. However, our cohort had a lower proportion of patients with septic shock and we are aware of the literature that suggests a greater benefit in this group of patients. This is why in our article, we mentioned this as a limitation and recommended a cautious interpretation, specifically for this subgroup of patients, since we do not rule out a benefit of early administration of antimicrobials in them.

The third aspect mentioned by the authors is related to confounding by indication. We agree that, in clinical practice, the most severe patients tend to be treated earlier. Taking into account the limitations in the area, we believe that one of the strengths of our analysis was to simulate the conditions of a clinical trial through a propensity score, a statistical technique that favors the reduction of bias. We clarified that we took into account the blood pressure and the severity of the patients as confounding factors and, among other things, we controlled them through the Sequential Organ Failure Assessment (SOFA) score and its cardiovascular score, as well as important covariates such as intravenous fluid use and lactate. These variables were balanced through the propensity score and we performed a sensitivity analysis by means of logistic regression models. In addition, we performed a subgroup analysis in which we included patients with septic shock and used two definitions with different hemodynamic parameters. For all of these models, both the crude mortality rate and that adjusted suggest the same conclusions.

We are appreciative to Dr. Jouffroy and Pr. Vivien for their responses and insights, and we agree with their words about the controversial aspect of this field. Our reported analysis, as well as other evidence in the area, suggests that there is no association between early antimicrobial administration and in-hospital mortality in patients with suspected or confirmed sepsis. We look forward to further studies that contribute to and shed light on the subject of antimicrobial administration time and its association with outcomes in patients with sepsis and septic shock. Nevertheless, we stand firmly that it is not just about the time of administration of antimicrobials, but the early recognition and the comprehensive approach by means of which, clinically and with laboratory tools, the most severe patients are recognized.

## Data Availability

Not applicable
